# The CCR5 antagonist maraviroc exerts limited neuroprotection without improving neurofunctional outcome in experimental pneumococcal meningitis

**DOI:** 10.1038/s41598-022-17282-0

**Published:** 2022-07-28

**Authors:** Ngoc Dung Le, Marel Steinfort, Denis Grandgirard, Aleksandra Maleska, David Leppert, Jens Kuhle, Stephen L. Leib

**Affiliations:** 1 Neuroinfection Laboratory, Institute for Infectious Diseases, University of Bern, Bern, Switzerland; 2 Graduate School for Cellular and Biomedical Sciences (GCB), University of Bern, Bern, Switzerland; 3 Multiple Sclerosis Centre, Neurology, Departments of Head, Spine and Neuromedicine, Biomedicine and Clinical Research, University Hospital Basel and University of Basel, Basel, Switzerland; 4 Research Center for Clinical Neuroimmunology and Neuroscience (RC2NB), University Hospital and University of Basel, Basel, Switzerland

**Keywords:** Bacterial infection, Central nervous system infections

## Abstract

One-third of pneumococcal meningitis (PM) survivors suffer from neurological sequelae including learning disabilities and hearing loss due to excessive neuroinflammation. There is a lack of efficacious compounds for adjuvant therapy to control this long-term consequence of PM. One hallmark is the recruitment of leukocytes to the brain to combat the bacterial spread. However, this process induces excessive inflammation, causing neuronal injury. Maraviroc (MVC)—a CCR5 antagonist—was demonstrated to inhibit leukocyte recruitment and attenuate neuroinflammation in several inflammatory diseases. Here, we show that in vitro, MVC decreased nitric oxide production in astroglial cells upon pneumococcal stimulation. In vivo, infant Wistar rats were infected with 1 × 10^4^ CFU/ml *S. pneumoniae* and randomized for treatment with ceftriaxone plus MVC (100 mg/kg) or ceftriaxone monotherapy. During the acute phase, neuroinflammation in the CSF was measured and histopathological analyses were performed to determine neuronal injury. Long-term neurofunctional outcome (learning/memory and hearing capacity) after PM was assessed. MVC treatment reduced hippocampal cell apoptosis but did not affect CSF neuroinflammation and the neurofunctional outcome after PM. We conclude that MVC treatment only exerted limited effect on the pathophysiology of PM and is, therefore, not sufficiently beneficial in this experimental paradigm of PM.

## Introduction

Despite the availability of antibiotic treatment and vaccines, acute bacterial meningitis (BM) caused by *Streptococcus pneumoniae* results in high case fatality rates and long-lasting neurofunctional deficits^1^. In addition, increasing resistance to first line antibiotic treatment, serotype replacement and lack of efficient adjuvant therapies to control brain damage negatively affect the management of pneumococcal meningitis (PM). One-third of PM survivors suffer from long-term neurological sequelae including cognitive impairment, learning disability and hearing loss, which can affect the development and life quality, especially if acquired during infancy or childhood^2,3^.

Upon contact with pneumococci, brain-resident cells such as microglia and astrocytes are activated and release high amount of inflammatory signaling molecules (cytokines and chemokines) and effector molecules such as matrix metalloproteinases (MMPs) and reactive oxygen (ROS) and nitrogen species (RNS), promoting blood–brain barrier (BBB) leakage and leukocyte recruitment^4^. As a consequence, the central nervous system (CNS) is injured by an endogenous excessive inflammation, on top of that induced by bacterial derivates. The brain injuries induced by PM are characterized by (1) cortical ischemic necrosis^5^ and (2) apoptosis in the subgranular zone of hippocampal dentate gyrus, a structure linked to learning and memory^6,7^. In addition, hair cells and spiral ganglion neurons (SGNs) in the inner ear are affected, resulting in sensorineural hearing loss^8,9^.

Dexamethasone is currently the only recommended adjunctive therapy for PM^10^ but is not recommended for BM in general and for neonates up to 6 weeks^11^. Moreover, dexamethasone aggravated hippocampal apoptosis and reduced learning capacity in experimental pediatric PM^12^. Thus, it is important to evaluate alternative therapeutic approaches to improve the outcome of infants and young children suffering from PM.

Maraviroc (MVC) is a C–C chemokine receptor type 5 (CCR5) antagonist. It is used for the treatment of patients infected with R5-tropic HIV-1 as the virus binds to the co-receptor CCR5 on the host's immune cells for entry^13^. Apart from inhibiting HIV-1 entry into host cells, MVC application demonstrates beneficial effects in treatment of experimental inflammatory diseases^14^. CCR5 has been implicated in a wide range of inflammatory brain diseases because of its important role in the recruitment of immune cells within tissues and to the CNS^15^. Inhibition of CCR5 signaling by MVC reduced the development of disease in experimental autoimmune encephalitis (EAE) by attenuating infiltration of immune cells into the CNS and by reducing microgliosis and astrogliosis^16^. Further, in experimental models of stroke and traumatic brain injury (TBI), MVC application inhibited astrocytic reactivity. In addition, MVC promoted motor recovery after stroke and improved learning and cognition following TBI^17^. Interestingly, the authors reported in a previous work that *Ccr5* knockout in mice resulted in enhanced hippocampal learning and memory, while overexpression caused memory deficits^18^.

Since PM is also characterized by the recruitment of immune cells into the CNS and the activation of astrocytes and microglia, we hypothesized that adjuvant MVC reduces neuroinflammation by inhibiting CCR5 signaling and therefore exerts neuroprotective effects. In addition, chronic MVC application may improve neurofunctional outcome.

## Results

### Maraviroc reduces inflammation in vitro

**Figure 1:**
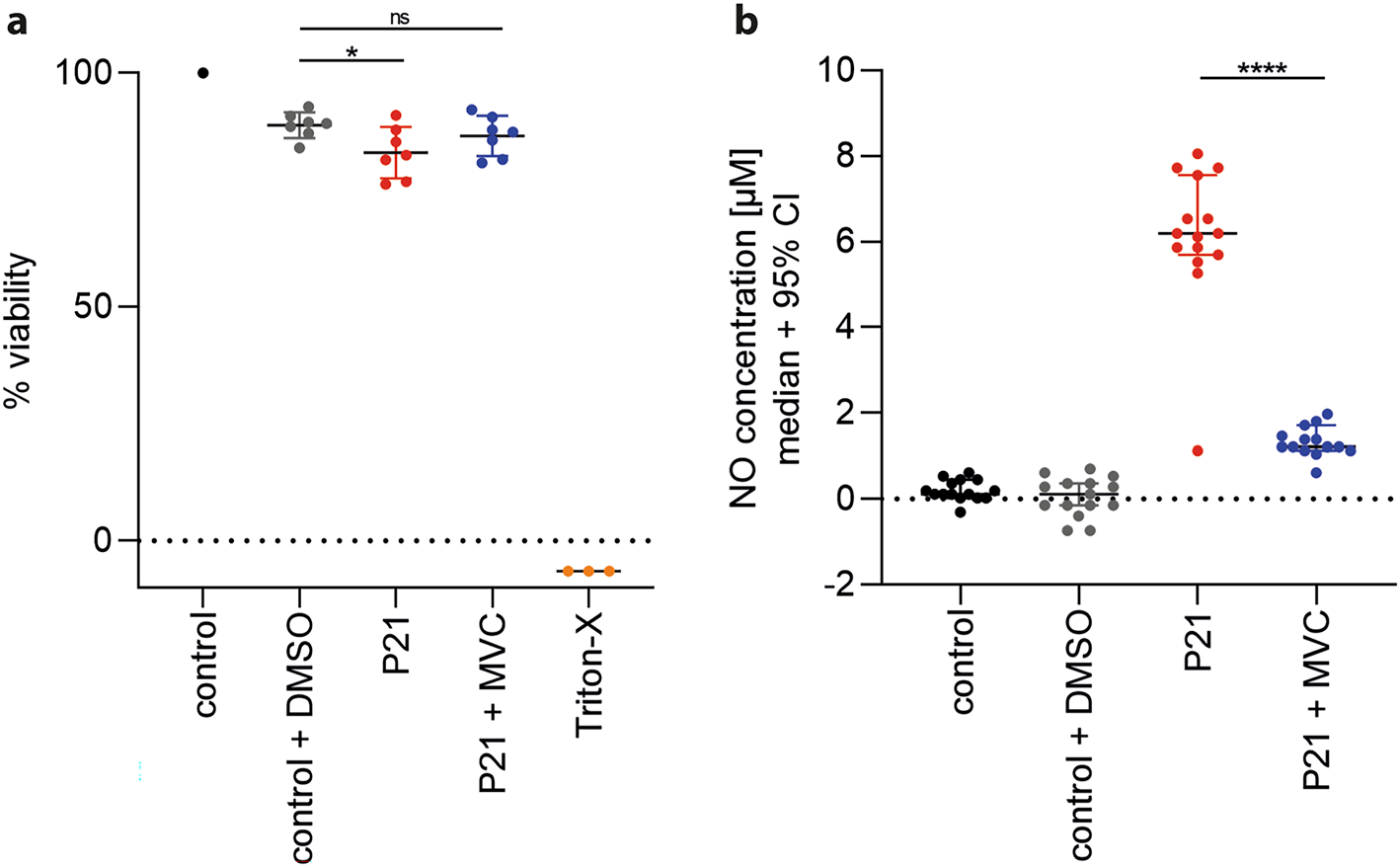
Effect of MVC in vitro. To determine whether MVC affected viability of the cells, the viability of the control group (black) was considered as 100% and used as reference for calculation. However, since MVC was dissolved in DMSO, we also assessed whether DMSO affected the viability negatively, as shown in gray (n = 7). 3% Triton-X (orange) (n = 3) was used a positive control for total cell mortality. (**a**) Results showed that P21 stimulation alone (red) (n = 7) (*p* = 0.02), but not with MVC treatment (blue) (n = 7), reduced cell viability compared to control group with DMSO. Further, MVC treatment significantly reduced NO concentration upon pneumococcal stimulation (*p* < 0.0001) (all groups n = 14–15) (**b**). Data are presented as mean ± SD if not stated otherwise. *p < 0.05, **p < 0.01, ***p < 0.001, and ****p < 0.0001 (Mann–Whitney test).

First, we determined whether MVC exerted cell toxicity and reduced inflammation in vitro by using primary rat astroglial cell cultures. Treatment of the culture with 100 µM MVC upon pneumococcal stimulation did not decrease viability (*p* = ns) compared to pneumococcal stimulation alone (*p* = 0.02), indicating that MVC did not exert direct toxicity (Fig. 1a). Further, we measured levels of NO as an index for inflammation. MVC significantly attenuated levels of NO upon exposure to living* S. pneumoniae* in comparison to stimulated, vehicle treated, cells (*p* < 0.0001) (Fig. 1b).

### Clinical parameters during acute PM

**Figure 2:**
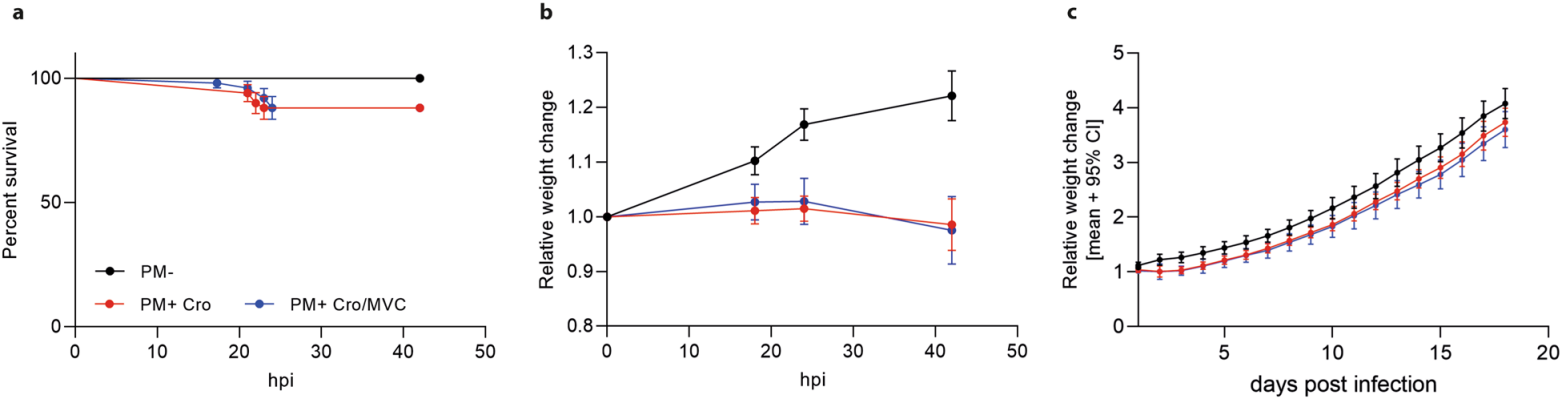
Survival and weight change in rats with PM. (**a**) Infected animals showed reduced survival compared to mock-infected ones independent of treatment modalities. Weight change during acute PM (**b**) and long-term recovery (**c**) was not affected by MVC application (PM- n = 16–20; PM + Cro n = 16–25; PM + Cro/MVC n = 16–25, depending on timepoints). Data are presented as mean ± SD.

Infected animals (PM+) treated either with ceftriaxone (Cro) and MVC or Cro monotherapy showed reduced survival (Fig. 2a) and weight loss (Fig. 2b) compared to mock-infected animals (PM-). Treatment with MVC in infected animals did not alter these parameters, neither during the acute phase (Fig. 2b) nor by chronic application (Fig. 2c), compared to vehicle treatment. The results indicate that chronic MVC application was well tolerated by the animals.

### Histological analyses during acute PM

**Figure 3:**
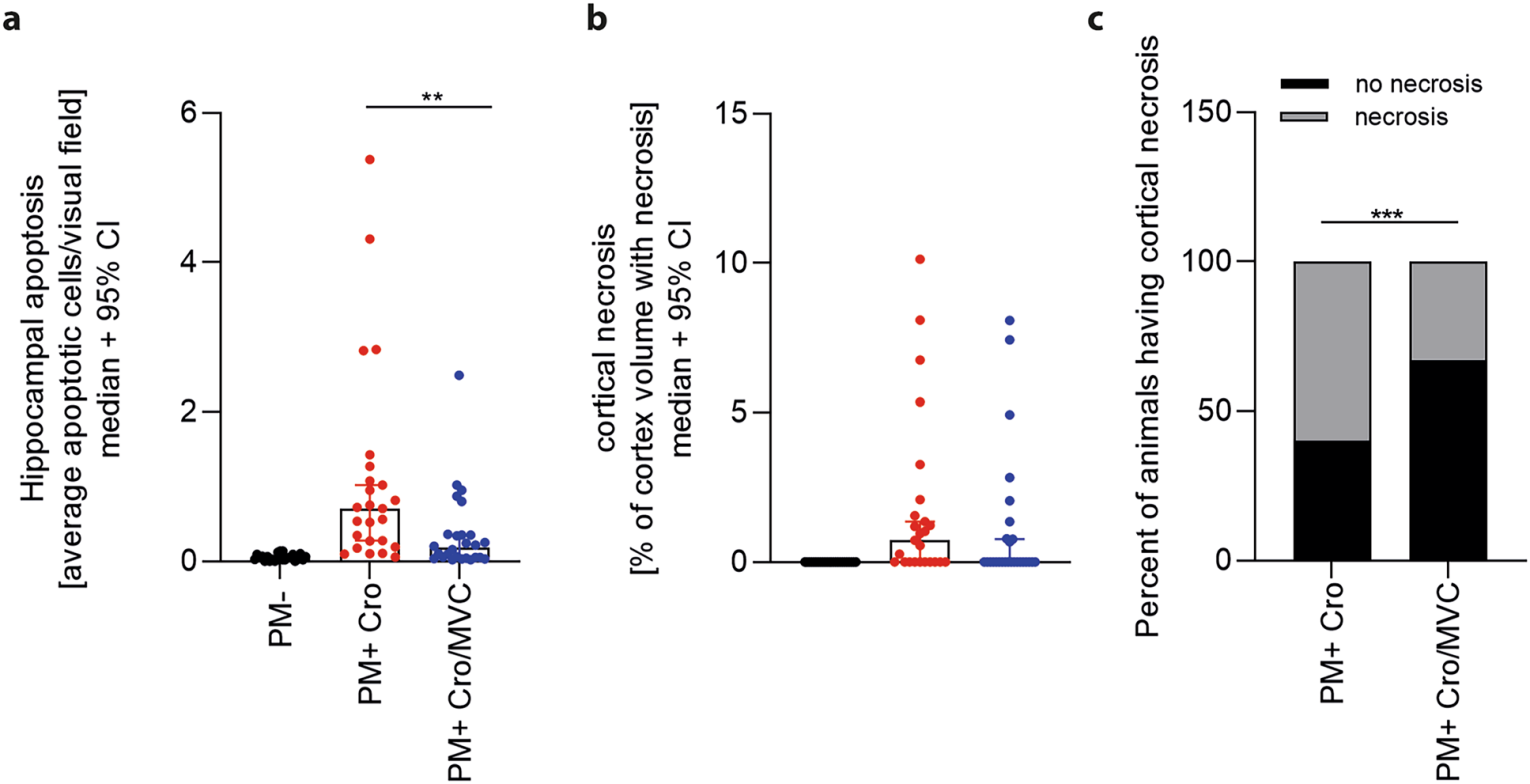
Histological analyses of rat brains during acute PM. (**a**) MVC treatment significantly reduced hippocampal apoptosis in infected animals compared to vehicle treatment, but not cortical necrosis (**b**) (PM- n = 24; PM + Cro n = 25; PM + Cro/MVC n = 26. (**c**) MVC reduced the number of infected animals displaying cortical necrosis. Data are presented as mean ± SD if not stated otherwise. *p* < 0.05, **p < 0.01, ***p < 0.001, and ****p < 0.0001 (Mann–Whitney test).

Histological analyses of the brain were performed to assess the neuroprotective effect of MVC. Apoptotic cells in the hippocampal dentate gyrus were significantly more numerous in infected animals as compared to mock-infected animals, despite Cro treatment. Infected animals treated in addition with MVC revealed significant lower number of apoptotic cells compared to those treated with Cro alone (*p* = 0.0011) (Fig. 3a). In contrast, the percentage of necrotic cortical volume in infected, Cro-treated animals was not reduced upon additional MVC treatment compared to vehicle (Fig. 3b). However, MVC treatment significantly reduced the proportion of animals exhibiting cortical necrosis (*p* = 0.0001) (Fig. 3c).

### MVC has no significant effect on neurofunctional outcome after PM

**Figure 4:**
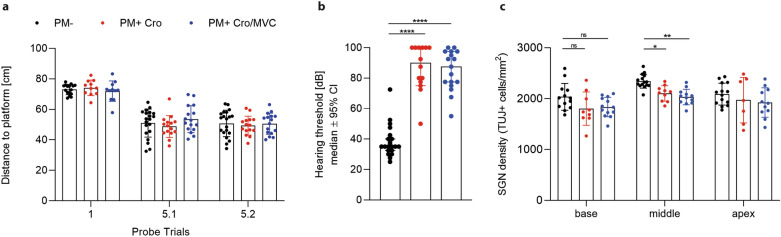
Neurofunctional outcome of PM. MVC treatment did not enhance learning/memory performance (PM- n = 16–20; PM + Cro n = 11–15; PM + Cro/MVC n = 11–16, depending on timepoints) (**a**) or improve hearing capacity (PM- n = 20; PM + Cro n = 14; PM + Cro/MVC n = 17) (**b**) in infected animals compared to vehicle treatment. Analysis of SGNs also revealed no reduced loss upon treatment with MVC (PM- n = 13–15; PM + Cro n = 7–10; PM + Cro/MVC n = 11–13, depending on timepoints) (**c**). Data are presented as mean ± SD if not stated otherwise. *p < 0.05, **p < 0.01, ***p < 0.001, and ****p < 0.0001 (Mann–Whitney test for hearing threshold; Two-way ANOVA for SGN).

Three weeks after infection, neurofunctional outcome after PM was evaluated using Morris water maze test for assessing learning and memory ability and hearing test for determining hearing threshold. Learning ability was observed in all groups, indicated by decrease of total distance swum until the platform was reached during the training trials over the consecutive five training days (data not shown). On day 5, spatial memory was assessed on the probe trials (D5.1 and D5.2) without platform. The average distance of the animal's swimming path to the virtual center of the platform location was not significantly different when comparing the paths of infected animals treated with MVC or vehicle (Fig. 4a).

Infected animals revealed increased hearing thresholds in response to broadband click stimuli compared to mock-infected animals (Cro-treated infected animals 90 dB vs. 35 dB in mock-infected animals, *p* < 0.0001; Cro/MVC-treated infected animals 87.5 dB vs. 35 dB in mock-infected animals, *p* < 0.0001) (Fig. 4b). In infected animals, MVC treatment did not significantly reduce click-induced hearing thresholds compared to vehicle treatment. In agreement with these data, infected animals revealed a trend towards decreased SGNs in basal and significant decreased SGNs in the middle turn compared to mock-infected animals (Fig. 4c). However, PM-induced loss of SGNs was not prevented by MVC treatment.

### Neurofilament light chain (NfL) is not affected by MVC

**Figure 5:**
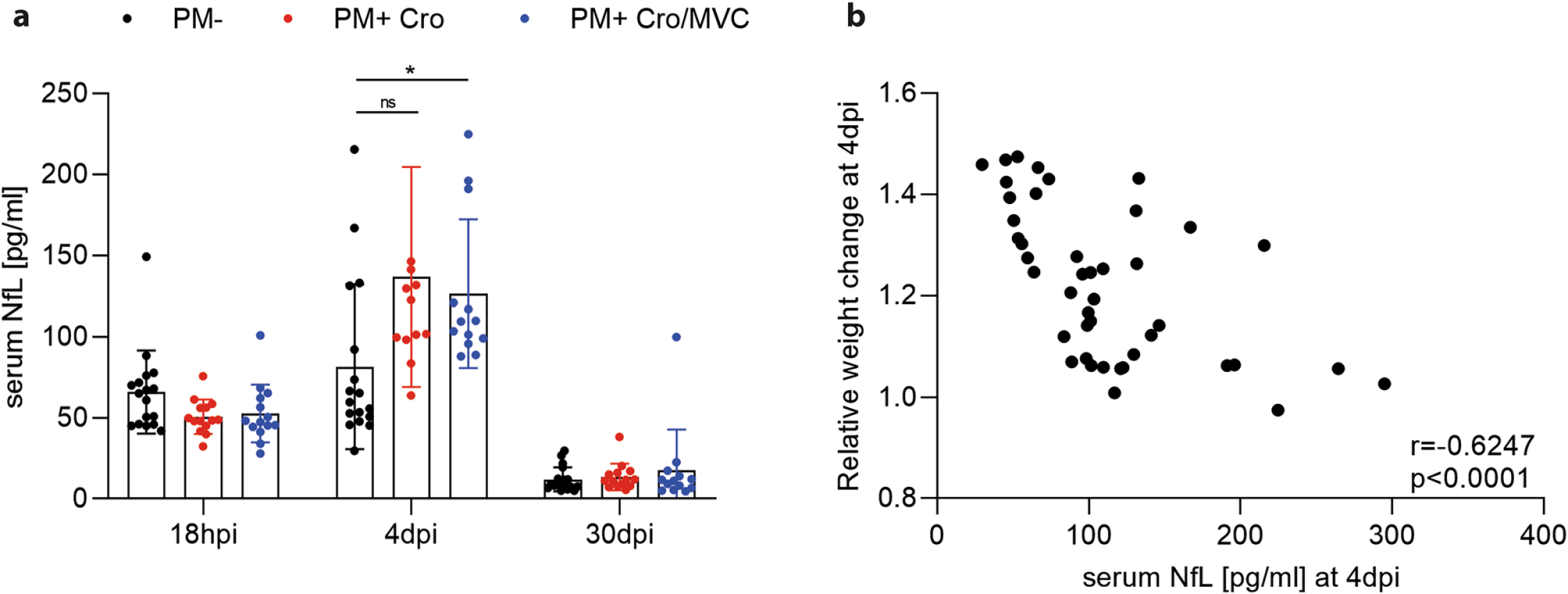
sNfL levels of animals at different timepoints during PM. (**a**) At 18 hpi, infected animals revealed similar sNfL levels compared to mock-infected animals. The sNfL levels of infected animals rose at 4 dpi and decreased again at 30 dpi to similar levels as found in mock-infected ones. MVC treatment did not affect sNfL at any given timepoints (PM- n = 17–18; PM + Cro n = 13–14; PM + Cro/MVC n = 13–14, depending on timepoints). (**b**) At 4 dpi, sNfL levels and relative weight change of the animals correlated (n = 43). Data are presented as mean ± SD. *p < 0.05, **p < 0.01, ***p < 0.001, and ****p < 0.0001 (Two-way ANOVA; Spearman correlation).

Blood was sampled at 18 hpi (at the time of the initiation of the therapy), 4 (representing the end of the acute phase) and 30 (after recovery of PM) dpi to assess the longitudinally course of serum NfL in PM and to determine the neuroprotective effect of MVC. At 18 hpi, mock-infected and infected animals showed similar levels of sNfL, in line with previous findings^19^ (Fig. 5a). At 4 dpi, infected animals revealed increased sNfL levels compared to mock-infected animals. However, when comparing MVC to vehicle treatment in infected animals, no difference in sNfL levels could be detected. At 30 dpi, all groups showed similar sNfL levels. Of note, sNfL levels at 4 dpi correlated with relative weight change of the animals at 18 hpi (r = − 0.4; *p* = 0.007) and 42 hpi (r = − 0.46, *p* = 0.0017) (data not shown). This correlation was even more pronounced at 4 dpi (r = − 0.62, *p* < 0.0001; Fig. 5b), indicating that disease severity, as determined by growth impairment, is associated with increased sNfL levels.

### Inflammatory parameters during acute PM are not affected by maraviroc

**Figure 6:**
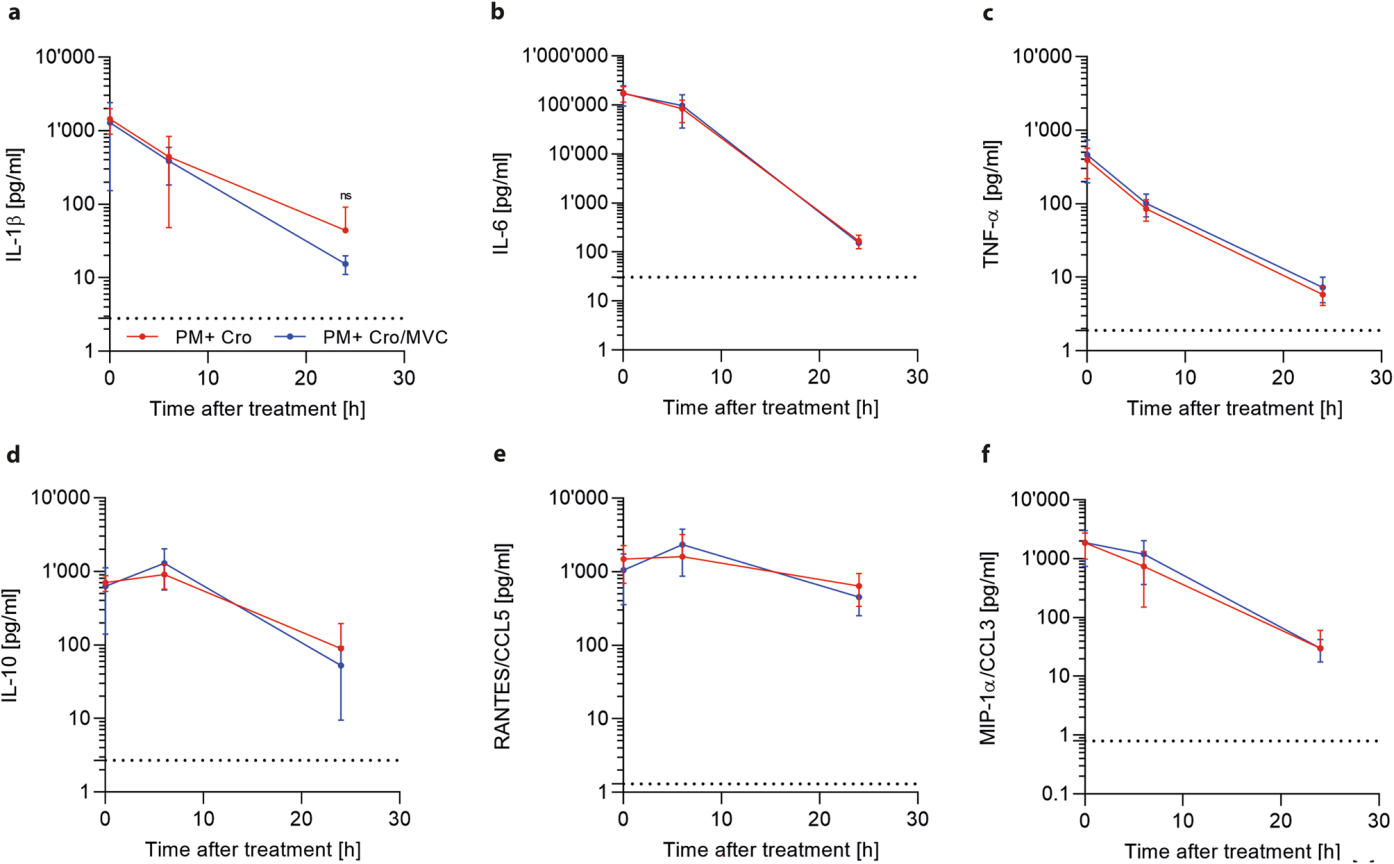
Inflammatory cytokines and chemokines during acute PM. (**a-f)** MVC treatment did not affect cytokine and chemokine levels at any given timepoints (PM + Cro n = 7–12; PM + Cro/MVC n = 7–13, depending on timepoints). Data are presented as mean ± SD. *p < 0.05, **p < 0.01, ***p < 0.001, and ****p < 0.0001 (Mann–Whitney test).

During the acute phase of PM, pro-inflammatory mediators are released by different brain-resident cells, resulting in CNS inflammation and neutrophil recruitment^4^. We measured the concentrations of several inflammatory parameters before treatment initiation (18 hpi) and at 6 h (24 hpi) and 24 h (42 hpi) later. Before treatment initiation, infected animals treated with MVC or vehicle showed comparable levels of CSF cytokines (IL-1β, IL-6, TNF-α, IL-10) and chemokines (MIP-α/CCL3, RANTES/CCL5). MVC treatment did not significantly alter the course of the cytokines/chemokines expression at later time points when compared to vehicle treatment (Fig. 6).

### MVC did not inhibit neutrophil recruitment to the CNS

**Figure 7:**
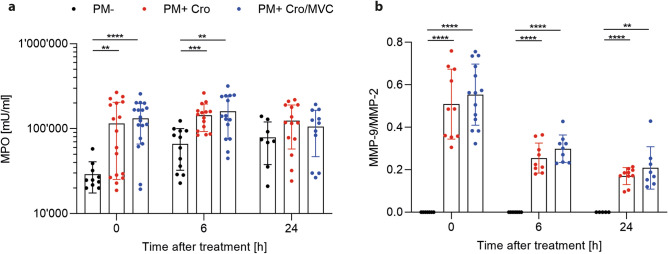
Neutrophil-associated inflammatory parameters. Myeloperoxidase (MPO) (PM- n = 8–12; PM + Cro n = 14–17; PM + Cro/MVC n = 11–18, depending on timepoints) (**a**) and matrix metalloproteinase 9 (MMP-9) (PM- n = 5–7; PM + Cro n = 9–10; PM + Cro/MVC n = 8–14, depending on timepoints) (**b**) were not affected by MVC treatment at any given timepoint. Data are presented as mean ± SD. *p < 0.05, **p < 0.01, ***p < 0.001, and ****p < 0.0001 (Two-way ANOVA).

Further, we assessed the levels of myeloperoxidase (MPO) and MMP-9 as a marker of neutrophilic recruitment in the CSF. Infected animals showed significant increased MPO levels before treatment initiation (0 h) and 6 h later compared to mock-infected animals, but not at 24 h (Fig. 7a). Similarly, MMP-9 levels were increased in infected animals at all three timepoints compared to mock-infected animals (Fig. 7b). MVC treatment did not reduce levels of either marker in infected animals compared to vehicle treatment.

### MVC does not affect microglial phenotype

**Figure 8:**
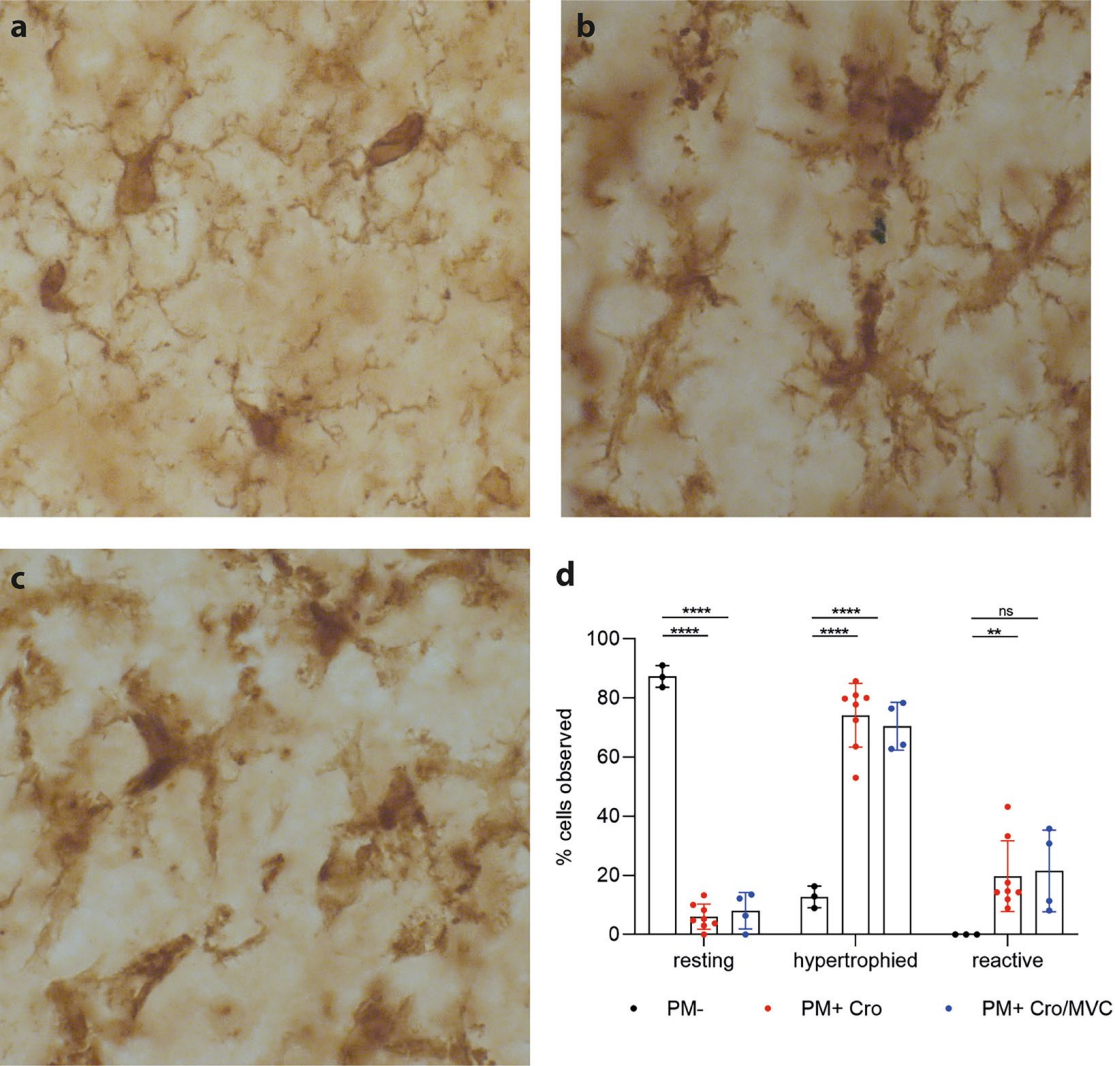
Microglial phenotype during acute PM. Microglial cells were classified into three categories: (**a**) resting, (**b**) intermediate/hypertrophied and (**c**) reactive. (**d**) MVC did not affect the phenotype of microglia during the acute phase of PM (PM- n = 3; PM + Cro n = 8; PM + Cro/MVC n = 4). Data are presented as mean ± SD. *p < 0.05, **p < 0.01, ***p < 0.001, and ****p < 0.0001 (Two-way ANOVA).

In addition, we assessed whether MVC treatment affected the morphology of microglial cells by Iba-1 staining. Mock-infected animals revealed more “resting” microglia, which are patrolling under physiological conditions, compared to infected animals (Fig. 8). Infected animals showed more microglia in hypertrophied and reactive state compared to mock-infected animals. Again, MVC treatment did not significantly modulate the morphology of microglia compared to vehicle treatment.

## Discussion

In the present study, we investigated the anti-inflammatory and neuroprotective effects of adjuvant MVC in PM. The results demonstrate that MVC treatment reduced the occurrence of cortical brain damage and exerted limited neuroprotective effect on hippocampal brain damage by reducing apoptosis. However, MVC treatment did not reduce neuroinflammation as reflected by the unaffected levels of CSF inflammatory parameters including cytokines/chemokines and neutrophilic markers. In addition, prolonged MVC treatment failed to improve the neurofunctional outcome of infected animals under the current experimental design.

Brain injury in acute PM involves the activation of CNS-resident brain cells and the recruitment of leukocytes to the CNS. The trafficking of leukocytes across BBB is mediated by chemokine receptors and their chemokines such as CCR5 and its ligands CCL3, CCL4 and CCL5^15,20^. Microglia and astrocytes are the main producer of chemokines in the CNS, leading to the recruitment and activation of CCR5^+^ leukocytes^21^. Leukocyte recruitment is essential for protecting against invading pathogens, but evidence has shown that excessive inflammation contributes to tissue damage in PM^22^.

MVC has been demonstrated to be effective in several experimental rodent models by inhibiting the recruitment of CCR5^+^ leukocytes to the site of injury and attenuating inflammation^16,17^. Nevertheless, the absence of neuroprotective effect of MVC has also been reported in progressive multifocal leukoencephalopathy (PML)^23^ and HIV^24^, where MVC did not improve the outcome of PML and did not impact CSF inflammatory markers in HIV patients.

Our in vitro results show that MVC did not exert toxicity on primary astroglial cells and reduced neuroinflammation induced by living *S. pneumoniae*. This anti-inflammatory effect of MVC has also been demonstrated in another study where MVC significantly reduced MMP-9 level upon exposure of cells to bacterial toxin^25^.

In a pilot study, we evaluated different concentrations of MVC (20, 50 and 100 mg/kg) in experimental PM (data not shown) and only found neuroprotective effects when given the highest concentration of MVC. Further, the dose of 100 mg/kg was previously shown to be beneficial during experimental stroke and TBI in adult mice^17^.

In the current experimental infant rat model of PM, the chosen dose (100 mg/kg) did not affect survival or weight gain of the animals during the acute or chronic phase of PM, indicating that MVC is well tolerated by the animals.

Inflammatory cytokines and chemokines as well as effector molecules such as MMPs produced by CNS-resident cells and infiltrating leukocytes limit the bacterial spread. However, they also contribute to increased BBB permeability and therefore brain damage^4^. In this experimental paradigm of PM, MVC treatment failed to reduce the CSF inflammation as reflected by the levels of cytokines/chemokines which were not decreased compared to vehicle treatment. Our findings are not in line with other studies, where MVC successfully reduced the levels of inflammatory cytokines/chemokines^26,27^. Of note, PM differs in its pathophysiology from other inflammatory diseases such as HIV infection or multiple sclerosis where the disease course is characterized by sustained chronic inflammation and participation of immune cells from the adaptive immunity compared to PM^28,29^. During the acute phase of experimental PM, levels of cytokines/chemokines and infiltrating neutrophils already peak at 18–24 hpi and decrease to baseline at 42–48 hpi^19,30,31^. This represents a very short therapeutic window where the adjuvant must act immediately to attenuate acute inflammation. Even with a treatment consisting of three doses of MVC (100 mg/kg) every 8 h during the acute phase, we were not able to modulate inflammation (data not shown).

Neutrophils are rapidly recruited at the site of infection for combatting the invading pathogens^31^. As a response, they secrete MMPs and nitric and oxygen species, also known to contribute to neuronal damage^4^. In the present study, CSF levels of myeloperoxidase (MPO), an enzyme expressed in primary/azurophilic granules of neutrophil granulocytes, were not reduced at any given timepoints, indicating that neutrophil recruitment to the CNS was not inhibited by MVC. Selectively blocking CCR5 may not be sufficient, since other receptors like the CXC chemokine receptor CXCR2 are critically involved in the migration of neutrophils toward inflammatory areas^32,33^. In line with this, CCR5 deficiency did not affect or even increase neutrophil recruitment in experimental models of tick-borne and herpetic encephalitis^34,35^. CCR5 also promotes degranulation (such as MMP release) and ROS production upon neutrophil extravasation^32^. Therefore, it could be expected that blocking CCR5 would attenuate degranulation, resulting in lower levels of MMP-9. However, in the current study, MMP-9 level was not decreased by MVC. Notably, MMP-9 is not only secreted by neutrophils but also by brain-resident cells including microglia, astrocytes and endothelial cells^4^. In addition, other chemokine receptors such as CXCR1, CCR1 and CCR2 also promote neutrophil degranulation and ROS production, so that exclusively blocking CCR5 may not have been sufficient to prevent it^32^.

While we could not detect anti-inflammatory effects of MVC on the different parameters we investigated, reduced hippocampal apoptosis was observed after treatment. Karampoor et al. have shown that MVC treatment decreased expression of the pro-apoptotic factors Bax, caspase-3 and caspase-9 while increasing the anti-apoptotic factor Bcl-2 in EAE model, suggesting that MVC may mediate neuroprotection by directly interfering with the modulation of programmed cell death^16^. Another study also suggested the role of CCR5 as a death receptor in neuronal cell lineages^36^. Thus, this may explain why MVC reduced brain injury in the current study without modulating overall inflammation.

Cell death in the hippocampus is associated with impaired learning and memory performance^12,37^ whereas preservation of the dentate gyrus can prevent this sequela^38,39^. MVC was shown to improve learning and cognition following TBI^17^. In the present study, the learning capacity was statistically not impaired in infected animals, when compared to mock-infected animals. The most likely explanation for this observation is that disease severity was mild and resulted in lower hippocampal apoptotic cell levels and therefore subsequently reduced impaired learning and memory, compared to previous study^38^. This would preclude the detection of MVC's beneficial effect on learning and memory.

Further, MVC treatment failed to improve click-induced hearing thresholds compared to vehicle treatment. In accordance, quantification of SGNs did not demonstrate reduced cell loss upon treatment with MVC. Since MVC was unable to reduce the levels of inflammatory cytokines/chemokines during acute PM, this might explain the lack of effect on hearing loss, as improvement by other adjuvant therapies in the present model usually relies on attenuated inflammation^38,40^.

Excessive inflammation mediated by host's immune response and bacterial toxins are the source for neuronal damage during PM^41^.

Neurofilament light chain is elevated during PM in patients^42^ and experimental rat model^19^ and can be used to evaluate drug therapy. In the current study, longitudinal assessment of neuronal damage was performed by determining sNfL levels to evaluate the neuroprotective effect of MVC. Results indicate that MVC failed to reduce the level of sNfL in this experimental paradigm. In particular, no difference between treatments could be detected at 4 dpi, a time point representing the end of the acute phase. This may seem contradictory to the fact that hippocampal apoptosis was attenuated at 42 hpi upon MVC treatment. Of note, histological assessment and sNfL determination were performed on two different cohorts (acute vs long-term) and at different timepoints (42 hpi for histological and 4 dpi for sNfL data). We hypothesize that sNfL concentration does not necessarily reflect this form of neuronal injury, but may rather be the consequence of cortical necrosis or other neuro-axonal damage not readily evidenced by cresyl violet staining. While necrotic cells release their content into the extracellular space, hippocampal apoptotic cells fragment into apoptotic bodies that are engulfed by resident glial cells. Doing so, NfL is unlikely to be released in extracellular space^43^. Further, PM induces apoptosis in hippocampal immature neurons^7^. Developing neurons first express nestin and vimentin (type VI and III intermediate filament proteins), which are then gradually replaced by NFs during development^44^. As a consequence, hippocampal apoptosis during PM would not contribute significantly to the elevation of sNfL levels. We also did not detect reduced cortical necrosis upon MVC treatment compared to vehicle treatment, that would then mostly explain why we did not observe effects of the therapy on sNfL levels. Nevertheless, the current results gave an insight into the longitudinally course of sNFL in experimental PM, which has not been described previously, with still elevated levels at 4 dpi in infected animals and then later return to basal level at 30 dpi. Further, results revealed that increased sNfL levels correlated with growth impairment, indicating that sNfL is a useful marker of disease severity.

Limitations of this study include the lack of analyzing the T lymphocytes. MVC has been shown to be particularly beneficial in diseases in which recruitment of T cells play a vital role^15^. Independent studies demonstrated an increase of CCR5^+^ T cells, especially CD8^+^ T cells, at the inflammation site and inhibition of CCR5 resulted in attenuated disease manifestation^45–47^. In contrast to the roles of neutrophils and monocytes which have been extensively described in PM, the function of T lymphocytes remains elusive. One study showed that lack of B and T cells recruitment upon induction of PM in mice resulted in impaired bacterial clearance and exaggerated systemic inflammatory responses^48^. In the present model, these manifestations could not be assessed as antibiotic treatment was applied to resolve the infection. Therefore, it remains unanswered whether lymphocyte recruitment was inhibited or still mediated by other than CCR5 chemotaxis.

Another limitation of this study is the lack of determining the concentration of MVC in the CSF. However, the chosen dose (100 mg/kg) for the current study is based on a published paper, which confirmed the presence of MVC in the CSF at a level, comparable to human therapeutic range^17^. Further, MVC is assumed to readily penetrate the CNS, a process which is even more enhanced when the BBB integrity is altered by inflammation^15^.

Taken together, the exact role of CCR5 in CNS infectious diseases remains difficult to determine, given the probably redundancy with other chemokine receptors. The specific function of CCR5 may depend on the infectious pathogen, as it can either be protective^49^, detrimental^47^ or having none effect on the disease course at all^50,51^.

## Conclusion

We demonstrate that blocking CCR5 with MVC in experimental PM did not attenuate neutrophil recruitment and brain-resident cell activation, as documented by unchanged expression levels of inflammatory parameters in the CSF. The results indicate that pharmacological blocking of CCR5 only exerted limited effect on the pathophysiology of PM and therefore CCR5-mediated activation may not be necessarily essential for defense against pneumococcal infection. Therefore, we conclude that MVC did not exert sufficient beneficial effect in this experimental paradigm of PM.

## Methods

### Infecting organism

A clinical isolate of *Streptococcus pneumoniae* P21 (serotype 3) was used as previously described^19,30^. Briefly, the bacterial isolate was cultured overnight in brain heart infusion (BHI) medium, diluted tenfold in fresh, prewarmed BHI medium and grown for 5 h to reach the logarithmic phase. The bacteria were centrifuged for 10 min at 3100 × *g* at 4 °C, washed twice and resuspended in saline (NaCl 0.85%). The bacterial solution was further diluted in saline to the desired optical density (OD_570nm_) and the inoculum concentration determined by serial dilution and culturing on Columbia sheep blood agar (CSBA) plates.

### Maraviroc delivery

For in vitro experiments, MVC (ViiV, Pfizer) was dissolved in DMSO (Sigma-Aldrich) and used at a concentration of 100 µM.

For in vivo experiments, MVC (100 mg/kg) was first dissolved in pure ethanol (Merck) and diluted tenfold in saline (NaCl 0.85%) containing 30% (2-hydroxypropyl)-β-cyclodextrin (Sigma-Aldrich). Vehicle treatment consisted of saline (NaCl 0.85%) containing 10% pure ethanol and 30% (2-hydroxypropyl)-β-cyclodextrin. Volume of vehicle was 100 µl/10 g bodyweight. Animals received treatment intraperitoneally (i.p.).

### In vitro astroglial cell stimulation

Primary astroglial cell culture consisting of astrocytes, microglia and oligodendrocytes were isolated from infant rat brains at postnatal day 3 (P3) (animal license BE 6/20) as previously described^30^. Astroglial cells were stimulated with living *S. pneumoniae* P21 (2.7 × 10^7^ CFU/ml) and treated with Cro (12 mg/ml, Rocephine, Roche). Cells were treated with MVC (100 µM) and compared to vehicle treatment (DMSO). Upon stimulation and treatment, cells were incubated for 24 h at 37 °C.

### Quantification of cell viability and nitric oxide (NO) production in vitro

After 24 h of stimulation, cell viability was assessed using an XTT assay, according to manufacturer’s instruction (Cell Proliferation Kit II, Sigma Aldrich, Buchs, Switzerland). Nitric oxide synthase activity was determined by measuring the release of NO_2_^−^ in the cell culture supernatant as previously reported^30^.

### Infant rat model of experimental PM

All animal studies were approved by the Animal Care and Experimentation Committee of the Canton of Bern, Switzerland (licenses no. BE 1/18 and 19/21). All experiments were performed in accordance with the guidelines and regulations approved by the Animal Care and Experimentation Committee of the Canton of Bern, Switzerland. The study was designed in accordance with ARRIVE guidelines. A well-established infant rat model of PM was used^30,52^. Wistar rat pups of mixed sex together with their dams were purchased from Charles Rivers (Sulzfeld, Germany). The dams were provided with tap water and pellet diet *ad libitum*. Litters were kept in rooms at controlled temperature of 22 ± 2 °C. During the acute phase of the disease (≤ 42 h post infection [hpi]), animals were housed in a laboratory with natural light. After this time, the animals were transferred to individually ventilated cages (IVC) in a room with controlled 12 h light/dark cycles. Infections were performed on 11-day old pups by intracisternal injection of 10 μl of the inoculum containing 1 ± 0.74 × 10^6^ CFU/ml of living *S. pneumoniae* serotype 3. Mock-infected animals received an equivalent volume of saline. To confirm PM, 5 µl of CSF was collected by puncturing the cisterna magna at 18 hpi, followed by serial dilution and cultivation on CSBA plates.

A total of 126 infant rats were included in this study. 56 animals were investigated during acute PM to assess neuroinflammation and brain damage (endpoints at 42 hpi) and 70 animals were used to investigate neurofunctional outcomes three weeks after infection. Infected and mock-infected animals were randomized for treatment with a single dose of adjuvant MVC (100 mg/kg, i.p.) and/or Cro (100 mg/kg in saline s.c., twice daily). Animals with Cro monotherapy received an i.p. vehicle treatment. MVC and Cro therapy were initiated at 18 hpi. Animals were weighted and clinical scored according to the following scoring scheme (1 = coma, 2 = does not turn upright, 3 = turns upright in > 5 s, 4 = turns upright in < 5 s, 5 = normal) at 0, 18, 24, and before sacrificing at 42 hpi. Spontaneous mortality was documented and animals without confirmed PM (CSF bacterial titer < 1 × 10^6^ CFU/ml) were excluded (n = 9). CSF samples at 18, 24, and 42 hpi were obtained by puncturing the cisterna magna using a 30-gauge needle. CSF samples were centrifuged (16,000 × *g*, 10 min, 4 °C) and supernatants were stored at − 80 °C for later analysis.

For long-term experiments, blood was sampled by puncturing the facial vein using a 20-gauge needle at 18 hpi, 4 days post infection (dpi) and terminally from the heart ventricle at 30 dpi. Blood (< 10% of total blood volume) was collected in Microvette® 200 Z (clotting activator/serum) (Sarstedt), kept at room temperature for at least 30 min and then centrifuged (10 min, 13,000 × g, 4 °C). The supernatant (serum) was collected and kept at − 80 °C for later analysis. For chronic application, animals received single dose of MVC or vehicle daily for additional two weeks. For both treatment groups, Cro application was continued for four days. Animals were sacrificed with pentobarbital (Esconarkon®, 150 mg/kg, i.p., Streuli Pharma AG, Switzerland) and perfused with 4% paraformaldehyde (PFA, Merck) in phosphate-buffered saline (PBS). Cochlea were harvested and fixed in 4% PFA for immunohistological analysis.

### Analysis of cytokine and chemokine expression in CSF

Cytokines and chemokines (IL-1β, IL-6, TNF-α, IL-10, RANTES and MIP-1α) were assessed using magnetic multiplex assay (Rat Cytokine/Chemokine MagneticPanel, Milliplex® Map Kit, Merck) on a Bio-Plex 200 station (Bio-Rad Laboratories) as previously described^19,30^. If the concentration of the sample was below the detection limit, a value corresponding to the detection limit provided by the manufacturer was used, considering the dilution factor. Detection limit for the samples were TNF-α 1.9 pg/mL, IL-1β 2.8 pg/mL, IL-10 2.7 pg/mL, IL-6 30.7 pg/mL, RANTES 1.3 pg/mL and MIP-1α 0.8 pg/mL.

### Analysis of myeloperoxidase activity in CSF

The activity of myeloperoxidase (MPO) in CSF was assessed as a parameter of neutrophilic activity. Five microliters of uncentrifuged CSF was used to measure the activity as described earlier^53^.

### Analysis of MMP-9 in CSF

The level of MMP-9 in CSF was determined by gelatin gel zymography as described earlier^19,54^. Expression of MMP-9 was determined as percentage of the constitutively expressed MMP-2 for each sample. Investigators were blinded to treatment modalities of animals when analyzing the levels of MMP-9.

### Histomorphometric analysis of cortical damage and hippocampal apoptosis

Neuronal damage in the cortex and the dentate gyrus of the hippocampus were quantified in all animals sacrificed at 42 hpi as previously described^30,38,55–57^. Investigators were blinded to treatment modalities of animals when performing histological assessments.

### Staining and categorical analysis of microglia morphology

Brains collected at 42 hpi were sampled into 45 µm free-floating cryosections and stained with Iba-1 for microglia as previously described^58^. Images of microglia in the cortex were randomly sampled under × 400 magnification from each animal (three sections per animal). Cells were classified into three categories, as previously described^58^: resting = round, oval body with thin and long processes; intermediate/hypertrophied = enlarged, darkened cell body with thick processes and less branching; reactive = enlarged, darkened cell body with little or no processes. The percentage of the different categories was calculated by first counting the total microglial cell number in the image followed by counting the three states of the microglia. Investigators were blinded to treatment modalities of animals when performing the analysis.

### Performance of Morris water maze to assess learning and memory capacity

Three weeks after infection, learning and memory capacity of animals was evaluated using Morris water maze (MWM) and video tracking system EthoVision XT-11 (Noldus Information Technology, Wageningen, Netherlands) as reported earlier^38^. Shortly, animals had to learn and memorize over five consecutive training days where the hidden platform is located in a tank filled with nontoxic black-colored water. Extra-maze distal cues were hung on the walls surrounding the arena for orientation. On the last training day (D5), probe trials without the platform were performed before (D5.1) and after the training session (D5.2) to determine long-term and short-term memory. Mean distance of rats to center of the virtual platform was evaluated. Investigators were blinded for treatment modalities of the animals.

### Assessment of hearing capacity by measuring auditory brainstem response

Four weeks post infection, auditory brainstem responses (ABR) were performed to determine the hearing capacity of the animals, as described previously^9,30,38^. Click recordings were performed with the Intelligent Hearing Systems SmartEP system. Hearing thresholds were analyzed by investigators blinded to treatment modality.

### Immunohistological analysis of spiral ganglion neuron density

Five weeks post infection, the cochlea was dissected for immunohistological analysis of SGN density as reported previously^30^. Analyses were performed by investigators blinded to treatment modality.

### Analysis of neurofilament light chain in serum

Neurofilament light chain (NfL) in serum was analyzed using the Nf-light SIMOA® immunoassay kit (Quanterix Corporation, Billerica, MA, USA), as previously described^19,59^. The antibody pair of this assay is full cross-reactive with murine NfL. Serum NfL was measured at 1:16 fold dilution by operators blinded for the data.

### Statistical analysis

Statistical analyses were performed with GraphPad Prism (Prism 7; GraphPad Software Inc., San Diego, USA). Since mock-infected animals treated with MVC or vehicle showed no significant differences (data not shown), we combined into one group (PM-). Infected animals are presented as PM + Cro or PM + Cro/MVC, meaning they were treated either with ceftriaxone combined with vehicle or ceftriaxone combined with MVC.

### Ethics approval and consent to participate

All animal studies were approved by the Animal Care and Experimentation Committee of the Canton of Bern, Switzerland (licenses no. BE 1/2018, 19/2021 and BE 06/20).

## Supplementary Information

Supplementary Information.

## Data Availability

The datasets used and/or analyzed during the current study are available from the corresponding author on reasonable request.
