# Supplementary material for: The CCR5 antagonist maraviroc exerts limited neuroprotection without improving neurofunctional outcome in experimental pneumococcal meningitis
Source: Sci Rep. 2022 Jul 28;12:12945. doi: 10.1038/s41598-022-17282-0 (PMC9334283; doi:10.1038/s41598-022-17282-0)
Supplement: Supplementary file 1 — Supplementary Information. [file 41598_2022_17282_MOESM1_ESM.pdf]

The CCR5 antagonist maraviroc exerts limited neuroprotection without improving neurofunctional outcome in experimental pneumococcal meningitis

Ngoc Dung Le[*1,2], Marel Steinfort[*1], Denis Grandgirard[1], Aleksandra Maleska Maceski[3,4], David Leppert[3,4], Jens Kuhle[3,4] and Stephen L. Leib[#1]

[1] Neuroinfection Laboratory, Institute for Infectious Diseases, University of Bern, Switzerland

[2] Graduate School for Cellular and Biomedical Sciences (GCB), University of Bern, Switzerland

[3] Multiple Sclerosis Centre, Neurology, Departments of Head, Spine and Neuromedicine, Biomedicine and Clinical Research, University Hospital Basel and University of Basel, Basel, Switzerland

[4] Research Center for Clinical Neuroimmunology and Neuroscience (RC2NB), University Hospital and University of Basel, Switzerland

[*]these authors contributed equally

[#]corresponding author

Short title: CCR5 antagonist maraviroc in experimental pneumococcal meningitis

Corresponding author:

Stephen L. Leib, Institute for Infectious Diseases, University of Bern

Friedbühlstrasse 51, 3001 Bern, Switzerland

stephen.leib@ifik.unibe.ch

Tel +41 31 632 49 49

Supplementary Figure

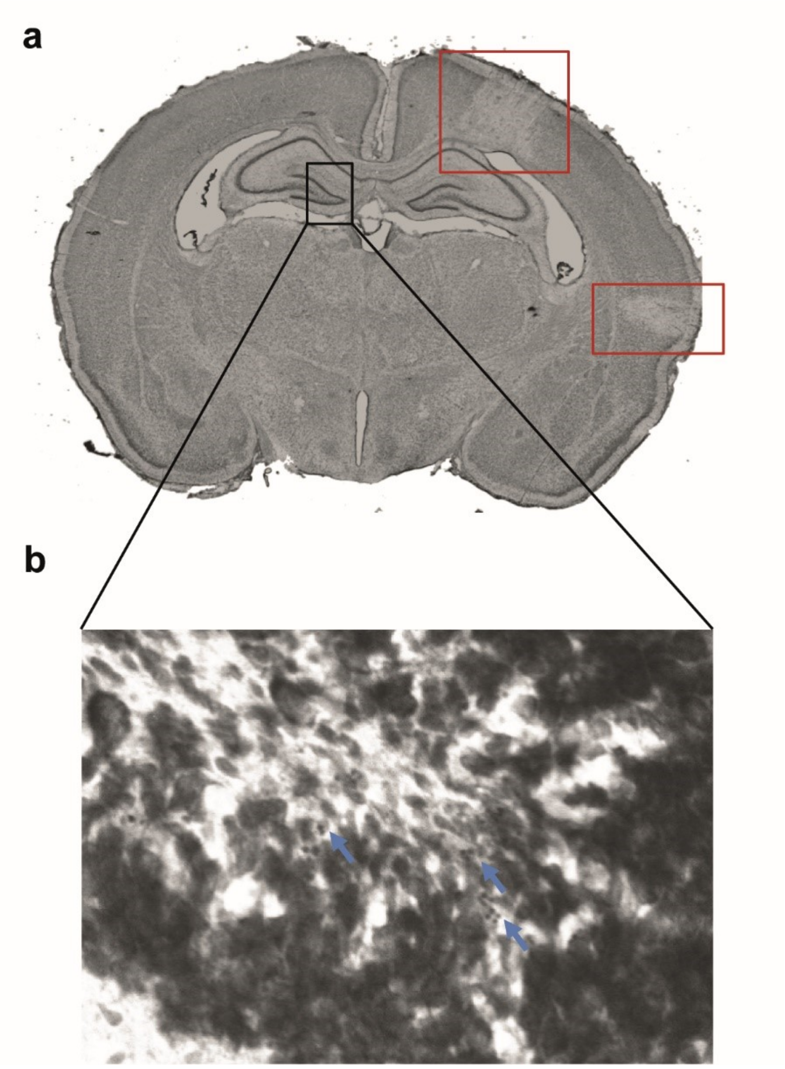

Supplementary Figure legend

Supplementary Fig. 1: Histological analyses of brain damage during acute PM. (**a**) Necrotic cell death can be found in the cortex as indicated by red squares. (**b)** Apoptotic cell death in the hippocampal dentate gyrus can be detected (blue arrows).
